# Correlating lesion size and location to deficits after ischemic stroke: the influence of accounting for altered peri-necrotic tissue and incidental silent infarcts

**DOI:** 10.1186/1744-9081-6-6

**Published:** 2010-01-19

**Authors:** Lisa D Alexander, Sandra E Black, Fuqiang Gao, Gregory Szilagyi, Cynthia J Danells, William E McIlroy

**Affiliations:** 1 Heart and Stroke Foundation Centre for Stroke Recovery, ON, Canada; 2 Sunnybrook Health Sciences Centre Research Institute, Toronto ON, Canada; 3 University of Toronto, Toronto ON, Canada; 4 L.C. Campbell Cognitive Neurology Research Unit, Toronto ON, Canada; 5 Department of Medicine (Neurology) at Sunnybrook Health Sciences Centre & University of Toronto, Toronto ON, Canada; 6 Department of Kinesiology, University of Waterloo, Waterloo ON, Canada

## Abstract

**Background:**

Investigators frequently quantify and evaluate the location and size of stroke lesions to help uncover cerebral anatomical correlates of deficits observed after first-ever stroke. However, it is common to discover silent infarcts such as lacunes in patients identified clinically as 'first-ever' stroke, and it is unclear if including these incidental findings may impact lesion-based investigations of brain-behaviour relationships. There is also debate concerning how to best define the boundaries of necrotic stroke lesions that blend in an ill-defined way into surrounding tissue, as it is unclear whether including this altered peri-necrotic tissue region may influence studies of brain-behaviour relationships. Therefore, for patients with clinically overt stroke, we examined whether including altered peri-necrotic tissue and incidental silent strokes influenced either lesion volume correlations with a measure of sensorimotor impairment or the anatomical localization of this impairment established using subtraction lesion analysis.

**Methods:**

Chronic stroke lesions of 41 patients were manually traced from digital T1-MRI to sequentially include the: necrotic lesion core, altered peri-necrotic tissue, silent lesions in the same hemisphere as the index lesion, and silent lesions in the opposite hemisphere. Lesion volumes for each region were examined for correlation with motor impairment scores, and subtraction analysis was used to highlight anatomical lesion loci associated with this deficit.

**Results:**

For subtraction lesion analysis, including peri-necrotic tissue resulted in a larger region of more frequent damage being seen in the basal ganglia. For correlational analysis, only the volume of the lesion core was significantly associated with motor impairment scores (r = -0.35, *p *= 0.025). In a sub-analysis of patients with small subcortical index lesions, adding silent lesions in the opposite hemisphere to the volume of the index stroke strengthened the volume-impairment association.

**Conclusions:**

Including peri-necrotic tissue strengthened lesion localization analysis, but the influence of peri-necrotic tissue and incidental lesions on lesion volume correlations with motor impairment was negligible barring a small index lesion. Overall, the potential influence of incidental lesions and peri-necrotic tissue on brain-behaviour relationships may depend on the characteristics of the index stroke and on whether one is examining the relationship between lesion volume and impairment or lesion location and impairment.

## Introduction

The size and anatomical location of stroke lesions is frequently evaluated to help advance our understanding of links between brain structure and human behaviour [1-4]. Accordingly, it is important for stroke neuroimaging analysts to have well-crafted methods for delineating and quantifying brain lesions that are visualized using widely available modalities such as magnetic resonance imaging (MRI). Lesion-based brain-behaviour studies in domains such as neuroscience and neuropsychology often involve patients with chronic stroke, most likely because it has been shown that no further lesion evolution typically occurs beyond one month after stroke [5,6]; this facilitates visualization of the full extent of structural tissue damage. Many lesion studies to date have also utilized T1-weighted MRI, perhaps because T1 images offer the best anatomical definition of lesions causing brain necrosis [7]. However, when utilizing MRI to visualize and quantify chronic stroke lesions, there is increasing uncertainty and debate concerning: 1) how to best define the boundaries of stroke lesions, as the necrotic core of many chronic stroke lesions is surrounded by tissue that blends in an ill-defined way into surrounding tissue, and 2) the extent to which pre-existing 'silent' infarcts may influence brain-behaviour relationships in patients exhibiting a first-ever clinically-overt stroke. With respect to the former, since a gold standard for lesion analysis does not exist - which may raise concerns about making comparisons between studies - investigators utilize a variety of manual, semi-automated, and automated methods for identifying and quantifying brain lesions. Multi- and single-parameter automated [8] or semi-automated thresholding or segmentation methods [9-11] are relatively common analysis techniques. While these methods may aid in determining the lesion boundary, the full extent of the lesion may not be captured for scans with a low image intensity or when there is a low contrast between brain tissue and the lesion [7]. Perhaps for these reasons, manual lesion tracing, which involves the use of computer software to trace the boundaries of a lesion on each slice of an MRI, continues to be a popular method of lesion identification [12].

**Figure 1:**
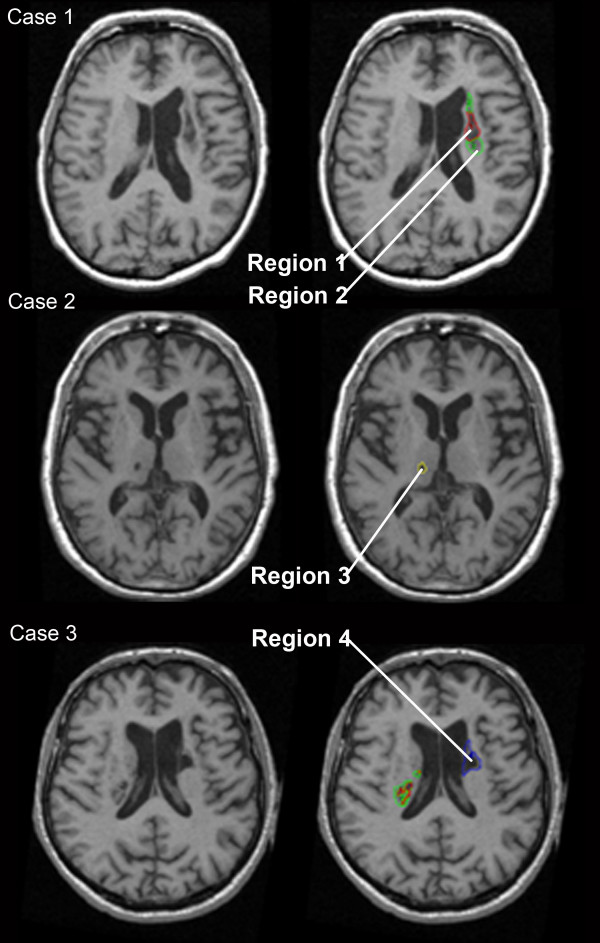
**Examples of the four regions used to denote differing categories of lesioned tissue**. Note that each row of MR images depicts examples from different patients. Region 1 represents the lesion core, Region 2 represents the peri-necrotic tissue, Region 3 represents lesions in the same hemisphere as the index stroke, and Region 4 represents lesions in the hemisphere opposite to Region 1.

When manually tracing lesions, defining the lesion boundary is challenging for infarcts that exhibit a well-defined necrotic core with poorly-defined bordering zones of peri-necrotic damage [13] (as demonstrated in Figure 1). In chronic stroke, this tissue likely represents varying degrees of gliosis and demyelination [14]. Whether or not one includes this bordering zone in lesion tracings could have a considerable impact on behavioural-neural correlates and on lesion volume measurements. Although it does not appear that this potential impact has been specifically investigated using T1-weighted MRI of chronic stroke, the influence of lesion boundary definitions on volume measurements has been examined using diffusion-weighted imaging (DWI) of acute stroke [15]. In a study of ischemic lesion volume measurements determined from DWI where one observer avoided tracing regions that were ill-defined and a second observer included less well-defined areas, the mean percent difference in volume measurements between the two observers was 93 ± SD 139% of the average lesion size (coefficient of variation 85 ± 130%) [15].

Brain-behaviour studies may be further complicated when remote, asymptomatic stroke is noted in addition to the primary lesion of interest. A recent study of incidental MRI findings in two-thousand persons from the general population (mean age of 63.3 years) found silent brain infarcts in 7.2% of participants, and found that the prevalence of such infarcts increased with age [16]. Also, the authors of the Cardiovascular Health Study examined more than three-thousand participants over age 65 without a known history of stroke, and found that 28% of persons had evidence of 'silent' infarcts [17]. Within 4 years, 7.3% of persons with silent infarcts in this group went on to have an overt stroke. Therefore, in patients identified clinically as first-ever stroke, it is likely common to note multiple stroke lesions, both lacunar and non-lacunar, either in both or in one hemisphere. However, probable asymptomatic lesions appear to have been generally disregarded or unaccounted for in brain-behaviour studies, which typically do not state whether or not patients with incidental ischemic lesions were excluded from study populations.

Overall, the extent to which computer-based lesion analyses of chronic stroke may be affected by the inclusion of remote strokes, lacunes or ill-defined peri-necrotic tissue is not known. We believe that how one defines the boundaries of the primary stroke and how one addresses secondary brain lesions will have an important impact on brain-behaviour relationships and lesion volume correlations with clinical measures after stroke. Therefore, using manual lesion tracing on 3D T1 MRI for a sample of chronic stroke patients with first-ever clinically overt stroke, the aim of the present study was to examine how subtraction lesion analysis and lesion volume correlations with a clinical measure were influenced by the inclusion of 1) ill-defined peri-necrotic tissue, 2) silent stroke lesions that were present in the same hemisphere as the clinically-overt stroke, and 3) silent lesions in the hemisphere opposite to the overt stroke. This investigation may help to guide neuroimaging analysts' decisions regarding the extent of stroke-related tissue damage to include in lesion analysis procedures for future lesion-based studies. Upper and lower extremity post-stroke impairment was chosen as our measure of interest, as limb hemiparesis is readily measureable and has been previously associated with injury in certain regions of the brain.

## Methods

### Study participants

A cross-sectional sample of consecutive participants in a research database who were identified clinically as first-ever hemispheric stroke, who presented with clinically-significant unilateral hemiparesis, and who underwent MRI scanning at 3 months post-stroke were analyzed in this study. Participants provided written informed consent to participate in research studies that were approved by the Research Ethics Board at Sunnybrook Health Sciences Centre. Each patient in our sample was drawn from the pool of participants from the Subacute Therapy with Amphetamine and Rehabilitation for Stroke (STARS) study [18]. Given that no additional benefit in motor or functional recovery was reported in this investigation for patients who received dextroamphetamine compared to placebo, patients were drawn from both treatment and control groups.

### Clinical measures of neurological and motor impairment

The National Institutes of Health Stroke Scale (NIHSS), which is a reliable and valid measurement tool [19], was used to characterize participants' acute stroke-related neurological deficits. The behaviour of interest examined for correlational and subtraction lesion analysis in this study was stroke-related motor impairment. Motor impairment was quantified using the motor domain of the Fugl-Meyer Assessment (FM), administered 3 months post-stroke by a physical therapist. The FM is reported to have excellent reliability and validity [20]. The FM motor domain is scored from 0 (flaccid hemiplegia) to 100 (normal movement), with 66 points allotted to the upper extremity and 34 points to the lower extremity. For computerized lesion analysis (subtraction analysis) based on the 3 month total Fugl-Meyer motor scores, patients (n = 41) were dichotomized into the following groups according to guidelines from available literature [21]: moderate hemiparesis ≥ 36 points, n = 30; severe hemiparesis >36, n = 11.

### Image acquisition

Anatomical imaging was performed at 3 months post-ictus on a dedicated research MRI scanner (1.5 T, GE Medical Systems, software version LX 8.2.5, NV/i hardware platform). High-resolution T1-weighted images were obtained using a standard three-dimensional fast spoiled gradient-echo anatomical imaging sequence (Repetition Time = 12.4 ms; Echo Time = 5.4 ms; Flip Angle, θ = 35°; Acquisition Matrix = 256 × 192; Slices = 124; Slice Thickness = 1.4 mm; Field of View = 22 × 16 cm).

### Lesion tracing

Manual image alignment, lesion tracing and lesion volume calculations were completed using ANALYZE 6.0 software. Tracing was performed with knowledge of patients' side of hemiparesis. Stroke lesions were visually identified, by a trained image analyst, as being hypointense compared to homologous contralateral tissue. The stroke lesions also did not exhibit mass effect, and MRI images were AC-PC aligned in order to facilitate contralateral tissue comparison. An experienced research neuroradiologist, who was blind to patient data and to the study's purpose, confirmed that the regions identified by the image analyst were in fact stroke-related lesions. The image analyst then traced the lesions from digital T1 images. Traced lesions were labelled as one of four regions:

(a) Region 1: Necrotic lesion core (isointense to cerebral spinal fluid - CSF)

(b) Region 2: Altered peri-necrotic tissue (hypointense, but not isointense to CSF)

(c) Region 3: Lesions in the same hemisphere as the index stroke

(d) Region 4: Stroke lesions in the other hemisphere

Examples of each Region are shown in Figure 1. Lacunes were differentiated from Virchow-Robin vascular spaces in accordance with recommended criteria [22]. For each patient, acute clinical stroke neuroimaging (usually CT) typically occurred as part of the standard of care for stroke. In instances where more than one non-lacunar stroke was observed in the hemisphere opposite to bodily hemiparesis, acute neuroimaging information (radiology reports, which were obtained for the STARS study) along with information on presenting clinical symptoms and probable stroke etiology was used to help distinguish the clinically-relevant stroke from pre-existing silent strokes. Both lesions were labelled as Region 1 if acute clinical information did not help to clarify which lesion, if either, was a remote stroke. When both a lacunar stroke and a non-lacunar stroke were present in the hemisphere opposite to bodily hemiparesis in regions that could reasonably be linked to motor impairment (i.e. sensorimotor cortex, basal ganglia, thalamus, corticospinal tract), unless the patient presented with a probable lacunar syndrome such as pure motor hemiparesis, the non-lacunar stroke was labelled as Region 1 and the lacunar stroke as Region 3. Lacunes were labelled as Region 1 in instances where no other lesions were present in the relevant hemisphere.

### Lesion analysis

Spatial normalization to the Montreal Neurological Institute (MNI) brain template was performed with lesion cost-function masking [23] and 16 non-linear iterations using the SPM2 software package for MATLAB. Region of interest (ROI) images were generated using MRIcro software, both individually and in a sequential manner such that the tracings for Region 2 were added to lesions identified as Region 1 to create the cumulative ROI of Region 1+2, and so on (i.e. Region 1: lesion core; Region 1+2: black core plus semi-altered peri-necrotic tissue; Region 1+2+3: core, peri-necrotic tissue and silent lesions same hemisphere as index stroke; Region 1+2+3+4: all lesions in both hemispheres). ROI images were transformed to the right hemisphere and were then subtracted to highlight regions that were frequently damaged in patients with severe hemiparesis and generally spared in patients with moderate hemiparesis. In other words, subtraction analysis was used to identify lesioned brain regions that were associated with motor impairment. (See Rorden & Karnath [12] for additional details concerning subtraction analysis.) The Talairach coordinates acquired in MRIcro were used to identify relevant anatomical structures implicated in the analyses [24]. ROIs were transferred to the right hemisphere because the relatively small number of patients in each group in a lateralized evaluation would have significantly reduced the power of lesion overlays. Also, the primary aim of this investigation was not to provide further evidence of the neuroanatomical localization of motor impairment, but to model the influence on brain-behaviour relationships of including ill-defined peri-necrotic tissue and silent infarcts in manual tracings of stroke. It should be noted that the association between side of brain lesion and FM scores was not significant (r = 0.06, *p *= 0.71).

### Statistical analysis

Statistical analyses were performed using SPSS version 15.0. Each variable was tested for normality using the Shapiro-Wilks test. Correlations between lesion volumes and Fugl-Meyer scores were determined using Spearman rank correlation coefficients. All *p*-values listed are two-tailed.

## Results

### Patient demographics and characteristics

A total of 41 patients were identified for analysis. Demographic data and stroke characteristics for included patients are available in Table 1. Among the 41 patients, 2 patients had two distinct strokes in the hemisphere contralateral to hemiparesis. Acute clinical data did not help to clarify the nature of these lesions (i.e. remote versus index stroke), so in these two patients both of the distinct lesions in the same hemisphere were labelled as Region 1. All other lesions that were present in the same hemisphere as the index stroke (labelled as Region 3) were lacunar strokes. Three patients, each of whom had moderate hemiparesis, exhibited a lacunar index stroke (Region 1).

**Table 1:** Patient demographic data and stroke characteristics

Severity of Motor Impairment:	Patients (n)	Fugl-Meyer Score:	Acute NIHSS Score	Average Age (years)	Sex (M)	Stroke Type (Isc, Hem, Lac)	Side of Index Stroke Lesion (R)
Moderate Impairment	30	72.5	7	71	15	26, 1, 3	16
Severe Impairment	11	27	12	64	7	11, 0, 0	5

'NIHSS' indicates the National Institutes of Health Stroke Scale. Median Fugl-Meyer and NIHSS scores are shown. 'Isc' indicates ischemic, 'Hem' indicates hemorrhagic, 'Lac' indicates lacunar, 'R' indicates right hemisphere

### Lesion volume measurements

The majority of patients exhibited relatively large lesions involving both cortical white matter and subcortical structures, and all but 3 patients exhibited some degree of tissue that was classified as Region 2. On average for the 41 patients, Region 1 accounted for 77.2% of total lesion volume (mean volume 15.9 cm^3^, SD 30.7) Region 2 for 19.6% (mean 4.1 cm^3^, SD 6.6), Region 3 for 0.28% (mean 0.058 cm^3^, SD 0.051) and Region 4 for 3.06% (mean 0.63 cm^3^, SD 1.1). Lacunes in the same hemisphere as the index stroke were noted in 29% of patients. Three patients with severe hemiparesis exhibited incidental lacunes. Also, 49% of patients had a lesion in the hemisphere opposite to the index stroke (5 of these patients were in the severe motor impairment group), and 24% of our 41 patients had both a lacune (Region 3) and a lesion in the hemisphere opposite to the index stroke (Region 4) in addition to their index stroke (Region 1). Lacunes accounted for 35% of the strokes in the hemisphere opposite to the index stroke (Region 4), while the lesions in 5% of these patients were relatively large and accounted for more than half of the total lesion volume.

### Statistical analysis of motor impairment measure and lesion volumes

As indicated in Table 2, the mean volume of the lesion core (Region 1) alone was significantly correlated to FM scores (r = -0.35, p = 0.025), and the volume of the peri-necrotic tissue showed a weak trend toward significant association with FM scores (Region 2; r = -0.26, *p *= 0.10). The association between motor impairment scores and the mean volume of lacunes did not reach statistical significance (r = 0.029, p = 0.86), nor did the association between motor impairment scores and the mean volume of lesions in the opposite hemisphere (r = 0.005, *p *= 0.98). As also indicated in Table 2, the association between FM scores and the cumulative lesion volumes was essentially identical between Region 1 (r = -0.35, p = 0.025), Regions 1+2 (r = -0.35, *p *= 0.028), Regions 1+2+3 (r = -0.34, *p *= 0.030), and Regions 1+2+3+4 (r = -0.34, *p *= 0.032).

**Table 2:** Association of motor impairment scores with the mean individual and cumulative lesion volumes for each region

Lesion Type:	Lesion Volume,^a ^cm^3^	Correlation Between Impairment Score and Lesion Volume (*p*-value)^b^
Region 1 ^c^	15.9 (30.7) 0.09 - 126.2	**-0.35** (0.025)*
Region 2 ^d^	4.1 (6.6) 0.0 - 28.5	**-0.28** **(0.10)**
Region 3 ^e^	0.058 (0.051) 0.0 - 0.17	**0.029** **(0.86)**
Region 4 ^f^	0.63 (1.1) 0.0 - 3.9	**0.005** **(0.98)**
Region 1 + 2	19.6 (36.7) 0.12 - 154.7	**-0.35** (0.028)*
Region 1 + 2 + 3	19.6 (36.2) 0.12 - 154.7	**-0.34** (0.030)*
Region 1 + 2 + 3 + 4	19.9 (36.6) 0.20 - 154.7	**-0.34** (0.032)*

Data for entire patient sample (n = 41) is shown

^a ^Lesion volumes are shown as mean (SD) and range

^b ^Correlation between mean lesion volume of a Region and Fugl-Meyer motor impairment scores (Values are Spearman rank correlation coefficients, with *p*-values in brackets)

^c ^Region 1 represents the lesion core

^d ^Region 2 represents the peri-necrotic tissue

^e ^Region 3 represents lesions in the same hemisphere as index stroke

^f ^Region 4 represents lesions in the opposite hemisphere

* Significant at *p *< 0.05

### Sub-analysis in patients with small subcortical lesions

Since the average volume of the lesion core was relatively large in this sample (15.9 cm^3^), we performed a sub-analysis to examine if the observations from our whole-group analyses were also seen in a subset of patients with small index lesions. Patients with 'small' lesions were defined as those with a core lesion volume that fell within the lower quartile (0.12 - 0.92 cm^3^) of core lesion volumes from whole-group data (n = 41). From our sample, we analysed 9 patients with small subcortical lesions where the mean volumes of Region 1 and Region 2 were 0.40 cm^3 ^and 0.34 cm^3 ^respectively. In other words, the mean total volume of the index stroke in this subset was ≤ 0.8 cm^3^. Of these 9 patients, 3 exhibited lacunar infarcts. The mean volumes of Region 3 and Region 4 were 0.038 cm^3 ^and 0.92 cm^3 ^respectively (see Table 3). Correlations between mean individual region volumes and FM scores for this group of 9 patients were as follows: Region 1, r = -0.58 (*p *= 0.05); Region 2, r = -0.22 (*p *= 0.3); Region 3, r = -0.64 (*p *= 0.03); and Region 4, r = -0.25 (*p *= 0.3). The association between FM scores and the cumulative lesion volumes in this group was: Region 1: r = -0.58, *p *= 0.05; Regions 1+2: r = -0.55, *p *= 0.06; Regions 1+2+3: r = -0.55, *p *= 0.06; and Regions 1+2+3+4: r = -0.62, *p *= 0.04.

**Table 3:** Association between mean lesion volumes and motor impairment scores in a sub-analysis of 9 patients with small subcortical lesions

Lesion Type:	Lesion Volume,^a ^cm^3^	**Correlation Between Lesion Volume & Impairment Scores (*p*-value) **^**b**^
Region 1	0.40 (0.24) 0.13 - 0.85	-0.58 (0.05)*
Region 2	0.34 (0.32) 0.03 - 1.04	-0.22 (0.3)
Region 3	0.038 (0.023) 0.0 - 0.064	-0.64 (0.03)*
Region 4	0.92 (1.4) 0.01 - 2.9	-0.25 (0.3)
Region 1 + 2	0.81 (0.41) 0.24 - 1.5	-0.55 (0.06)
Region 1 + 2 + 3	0.77 (0.43) 0.24 - 1.4	-0.55 (0.06)
Region 1 + 2 + 3 + 4	1.69 (1.74) 0.40 - 3.91	-0.62 (0.04)*

^a ^Lesion volumes are shown as mean (SD) and range

^b ^Correlation between mean lesion volume of a Region and Fugl-Meyer motor impairment scores (Values are Spearman rank correlation coefficients, with *p*-values in brackets)

* Significant at *p *< 0.05

### Subtraction lesion analyses

**Figure 2:**
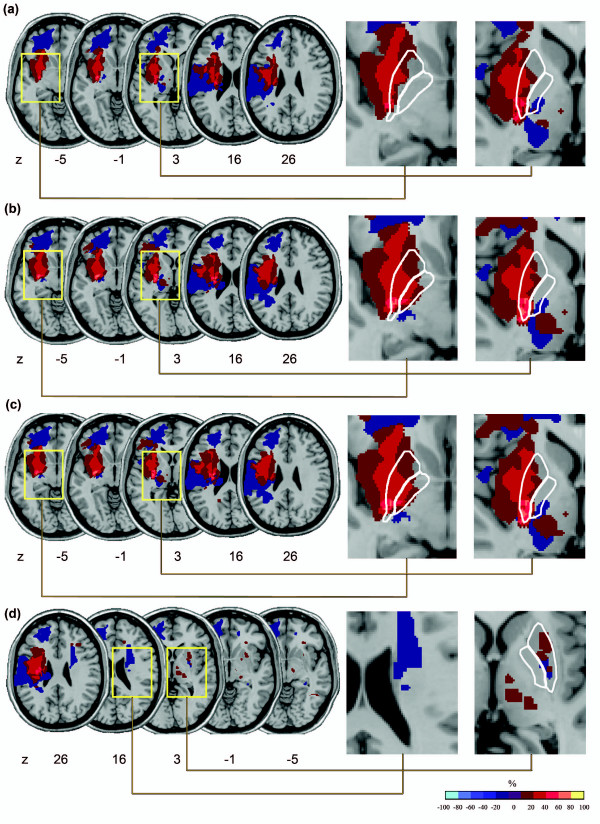
**Subtraction lesion analysis**. The lesion overlay of patients with mild to moderate motor impairment was subtracted from the lesion overlay of patients with severe motor impairment. (a) Region 1 only, (b) Region 1+2, (c) Region 1+2+3, (d) Region 1+2+3+4 (region 4 shown). The colours indicated in the key denote frequencies, where voxels that were more often damaged in severely affected patients and spared in mild to moderately impaired patients appear toward the yellow end of the spectrum. In the enlarged images, the lentiform is outlined in white. For subtraction analysis involving Region 1 (Figure 2a), a small region in the inferior posterior putamen was identified as being lesioned 40-60% more frequently in patients with severe motor impairment than in those with moderate impairment. However, in the subtraction analysis that included Regions 1+2 and Regions 1+2+3 (Figures 2 b,c), it is seen that a region spanning from the inferior to the superior aspect of the posterior putamen was lesioned 60-80% more frequently in patients with severe motor impairment than in those with moderate impairment. No regions in the opposite hemisphere were notably highlighted as more frequently lesioned in the severely impaired group (Figure 2d).

As seen in Figure 2, the inclusion of peri-necrotic tissue notably influenced subtraction analyses. For subtraction analysis using Region 1 (Figure 2a), a small area in the inferior posterior putamen was identified as being lesioned 40-60% more frequently in patients with severe motor impairment than in those with moderate impairment. However, in the lesion analysis that included Regions 1+2 and Regions 1+2+3 (Figures 2b, c), it was seen that a region spanning from the inferior to the superior aspect of the posterior putamen was lesioned 60-80% more frequently in patients with severe motor impairment than in those with moderate impairment. In the hemisphere ipsilateral to hemiparesis (i.e. analysis for Region 4), no regions were notably highlighted as more frequently lesioned in the severely impaired group (Figure 2d).

## Discussion

There is a need to clarify the influence of lesion boundary definitions and of the inclusion of incidental infarcts on lesion-based brain-behaviour studies. In general, stroke neuroimaging analysts lack standardized guidelines for how to address incidental infarcts and define lesion borders when manually tracing lesions [13]. While it appears that lesion-based studies to date have generally not accounted for tissue damage beyond the necrotic lesion core, no study to date has examined whether regions of additional injury could in fact have an impact on brain-behaviour relationships. Thus, we sought to investigate whether different lesion boundary definitions and the inclusion of silent infarcts influenced anatomical behavioural localization or lesion volume correlations with a clinical measure of motor impairment.

Our results showed that the volume of the necrotic lesion core (Region 1) was most closely associated with motor impairment scores. Although the volume of the peri-necrotic region showed a weak trend toward significant association with motor impairment scores, analysis of cumulative lesion volumes showed that the addition of the peri-necrotic tissue to the volume of the necrotic lesion core neither strengthened nor weakened the association between lesion volume and motor impairment scores for whole group or subgroup (n = 9) analyses. With regard to our subtraction lesion analysis, previous research suggests that the posterior putamen is involved in motor control [25-27]. Including the peri-necrotic tissue in our subtraction lesion analysis appeared to strengthen our results, as a larger region of more frequent putaminal damage was noted in our subtraction analysis once lesion tracings included the peri-necrotic region (seen in Figure 2).

In general, these results suggest that the correlation between lesion volume and post-stroke impairment may not be strongly influenced by the volume of the peri-necrotic region. However, it may be the location more so than the volume of the peri-necrotic tissue that is important in behavioural-anatomical relationships, since accounting for the volume of this region had an equivocal influence on lesion volume correlations with motor impairment, while accounting for peri-necrotic tissue notably strengthened the anatomical localization of motor deficits in our subtraction analysis. This finding regarding volume-impairment correlations may have been due to the fact that the average volume of the necrotic lesion core was 4 times larger than the volume of the peri-necrotic region, which could have dampened the influence of the peri-necrotic region on correlational analyses; however, including the peri-necrotic region may have impacted our lesion subtraction analysis because increasing the size of lesion tracings may increase the probability that overlap will be observed for lesions that fall within close proximity to one another.

A recommendation that peri-necrotic tissue be included in lesion tracings for lesion-based studies that involve procedures such as subtraction analysis is supported by previous literature, which suggests that this region may in fact remain dysfunctional in chronic stroke patients. For instance, in a functional MRI (fMRI) study of the activation observed in the cortical peri-infarct region during finger tapping, Cramer and colleagues [13] noted that even among well-recovered chronic stroke patients, activation in the area surrounding the core of the infarct was reduced compared to normal controls, implying that this region may remain dysfunctional post-stroke. Likewise, Seitz and colleagues [28] postulate that since persisting depression of cerebral metabolism and blood flow beyond the necrotic core of brain infarction has been noted, chronic structural brain lesions revealed on CT and MRI likely reflect only a portion of the total amount of brain tissue affected by ischemia. This evidence, which suggests that the peri-infarct region, and perhaps the region beyond the observable lesion boundaries, can remain dysfunctional in the chronic stage of stroke, lends support to the suggestion that it may be desirable to include the altered peri-necrotic tissue when tracing lesions for anatomical-behavioural localization studies. This view is supported by our subtraction lesion analysis findings.

The inclusion of pre-existing lacunes in the same hemisphere as the index stroke did not notably influence our subtraction lesion analysis - most likely because of their small volume - and the volume of lacunes was not significantly correlated to motor impairment scores, either alone or when added to cumulative lesion volumes. However, this latter finding may stem from the observations that only 29% of patients in our full sample (n = 41) exhibited lacunes, few patients in our sample had small index lesion volumes, and the average volume of the lesion core (Region 1) was substantially larger than Region 3, which may have dampened the potential association between lacunar lesion volumes and clinical measures. Previous investigations suggest that lacunes, which result from the occlusion of a penetrating cerebral artery [29,30], can have a significant impact on the clinical expression of disease. For example, the seminal work of Snowden and colleagues in The Nun Study suggested that lacunes may play a role in determining the presence and severity of Alzheimer Disease symptoms [31]. Also, the presence of lacunes is indicative of a higher total burden of cerebral small vessel disease, which is thought to be an important prognostic determinant after stroke [32]. Although lacunes have been noted to be important and relevant findings by previous investigators, lacunes did not have a notable influence in our investigation on lesion volume correlations with FM scores nor on subtraction lesion analysis. The use of varying MRI sequences between studies that have examined the clinical implications of lacunes could also be contributing to differences in findings among groups. The influence of lacunes on brain-behaviour relationships could perhaps also depend on the clinical measure of interest; for instance, the burden of lacunes could be more tightly coupled with measures of cognitive impairment than with motor impairment. Consequently, taking into consideration the findings from this investigation and from previous authors, while the inclusion of lacunes may not have a notable impact on lesion volume correlations with clinical measures nor on behavioural-anatomical localization findings, it is likely advisable to at least note the presence of lacunes in patients involved in such studies. As previous literature indicates, the presence of lacunes could be an important variable in predictive statistical analyses.

Lastly, the volume of lesions in the hemisphere opposite to that of the index stroke (Region 4) was not significantly associated with motor impairment scores for whole-group (n = 41) analysis and showed no appreciable overlap for lesion location on subtraction lesion analysis. This latter finding may be due, in part, to the relatively small size of these lesions in patients with severe hemiparesis in our sample. This suggestion highlights a shortcoming of the subtraction lesion analysis technique used in this study. Since patients are dichotomized into 'affected' and 'unaffected' groups for this analysis rather than being evaluated along a behavioural continuum, which is possible when using analysis techniques such as voxel-based lesion symptom mapping (VLSM) [33], data regarding the potential impact of lesions in both hemispheres may have been lost. Future investigations in this realm with a larger sample size may allow for VLSM analysis. The observation that pre-existing lesions in the opposite hemisphere were not associated with more severe motor impairment may also have been the result of the location of these lesions. For example, fMRI studies have revealed bilateral activation in the cerebellum, basal ganglia, and sensorimotor cortices during active limb movements [26], which suggests that activity in a distributed bi-hemispheric network is involved in limb motor control. But, if the lesions in our patient population fell outside of these regions associated with active limb movement, then the presence of lesions in both hemispheres may not have notably influenced our results. On the other hand, the results from our sub-analysis of patients with small subcortical lesions lends support to the idea that pre-existing asymptomatic stroke lesions in the cerebral hemisphere opposite to the hemisphere of a clinically overt stroke may indeed be clinically important. These results showed that the association between cumulative lesion volumes and motor impairment scores in our subset of 9 patients with small index strokes was strengthened when lesions in the opposite hemisphere were accounted for. From this portion of our analysis, the most important finding to note is likely that there appears to be variability with respect to the influence of incidental lesions in the opposite hemisphere on brain-behaviour relationships depending on the size of the index stroke.

## Conclusions and future directions

In view of the increasing number of studies that use computer-based tools for analyzing the size and location of lesions underlying post-stroke deficits in the many domains of brain function such as memory, learning, perception and sensorimotor function, it is important to better understand how what one defines as 'lesion' could influence the findings of such studies. Although exploratory, the results from this study suggest that the influence of peri-necrotic tissue and incidental lesions may depend on whether one is examining the relationship between lesion volume and impairment or the relationship between lesion location and impairment. The characteristics of the index stroke may also be an important determinant of the magnitude of influence associated with additional regions of stroke-related ischemic damage. It should be noted that our results can only be generalized to patients with hemiparesis; the behaviour or impairment under study could also be an important determining factor underlying the relationship between lesion characteristics and measures of behaviour, as other behaviours may be more heavily influenced by lacunes or contralateral lesions. A degree of caution is also important when ascribing pathological significance to areas of MRI signal abnormality, as the underlying etiology of these abnormalities cannot be determined for certain from MRI alone. Careful imaging-pathological comparisons are needed to understand this, and such studies have described gliosis and demyelination in the peri-infarct region. However, it is often not possible to infer whether any neural activity could still be supported by this perinfarct region. Accordingly, careful MRI analysis and quantification of the clinically relevant signs are essential when conducting lesion-based analyses of brain-behaviour relationships. Finally, for lesion-based studies involving computerized manual or automated lesion tracing, we also wish to emphasize the need for authors to explicitly describe lesion identification and analysis procedures (i.e. what was considered as 'lesion', how incidental findings were managed) in future study methodologies in order to facilitate comparisons between investigations.

## Competing interests

The authors declare that they have no competing interests.

## Authors' contributions

LDA co-conceived of the research question and study design, performed data analysis, interpretation and statistical analysis, and was the primary manuscript author. SEB was involved in formulating the research question and study design, and assisted with data interpretation and manuscript authorship. FG was involved in neuroimaging analysis and the interpretation of neuroanatomical data. GS devised our lesion analysis procedures. CJD assisted in data collection, interpretation, and manuscript review. WEM was involved in formulating the research question and study design, data analysis and interpretation, and assisted with authoring the manuscript. All authors read and approved the final manuscript.
